# Dynamic regional homogeneity alterations and cognitive impairment in patients with moderate and severe obstructive sleep apnea

**DOI:** 10.3389/fnins.2022.940721

**Published:** 2022-08-26

**Authors:** Kunyao Li, Yongqiang Shu, Xiang Liu, Wei Xie, Panmei Li, Linghong Kong, Pengfei Yu, Yaping Zeng, Ling Huang, Ting Long, Li Zeng, Haijun Li, Dechang Peng

**Affiliations:** ^1^Medical Imaging Center, The First Affiliated Hospital of Nanchang University, Nanchang, China; ^2^Science and Technology Division, Big Data Research Center, The Second Affiliated Hospital of Nanchang University, Nanchang, China; ^3^PET Center, The First Affiliated Hospital of Nanchang University, Nanchang, China

**Keywords:** dynamic regional homogeneity, support vector machine, obstructive sleep apnea, cognitive impairment, spontaneous brain activity

## Abstract

**Background and purpose:**

Previous studies have found that abnormal local spontaneous brain activity in patients with obstructive sleep apnea (OSA) was associated with cognitive impairment, and dynamic functional connections can capture the time changes of functional connections during magnetic resonance imaging acquisition. The purpose of this study was to investigate the dynamic characteristics of regional brain connectivity and its relationship with cognitive function in patients with OSA and to explore whether the dynamic changes can be used to distinguish them from healthy controls (HCs).

**Methods:**

Seventy-nine moderate and severe male OSA patients without any treatment and 84 HCs with similar age and education were recruited, and clinical data and resting functional magnetic resonance imaging data were collected. The dynamic regional homogeneity (dReHo) was calculated using a sliding window technique, and a double-sample *t*-test was used to test the difference in the dReHo map between OSA patients and HCs. We explored the relationship between dReHo and clinical and cognitive function in OSA patients using Pearson correlation analysis. A support vector machine was used to classify the OSA patients and HCs based on abnormal dReHo.

**Result:**

Compared with HCs, OSA patients exhibited higher dReHo values in the right medial frontal gyrus and significantly lower dReHo values in the right putamen, right superior temporal gyrus, right cingulate gyrus, left insula and left precuneus. The correlation analysis showed that the abnormal dReHo values in multiple brain regions in patients with OSA were significantly correlated with nadir oxygen saturation, the oxygen depletion index, sleep period time, and Montreal cognitive assessment score. The support vector machine classification accuracy based on the dReHo difference in brain regions was 81.60%, precision was 81.01%, sensitivity was 81.01%, specificity was 82.14%, and area under the curve was 0.89.

**Conclusion:**

The results of this study suggested that there was abnormal dynamic regional spontaneous brain activity in patients with OSA, which was related to clinical and cognitive evaluation and can be used to distinguish OSA patients from HCs. The dReHo is a potential objective neuroimaging marker for patients with OSA that can further the understanding of the neuropathological mechanism of patients with OSA.

## Introduction

Obstructive sleep apnea (OSA) is the most common sleep disorder, characterized by repeated collapse of the upper respiratory tract during sleep, resulting in the reduction or cessation of recurrent ventilation, which causes intermittent hypoxemia, hypercapnia, and sleep microarousal (Deegan and McNicholas, 1995). Previous studies have shown that the prevalence rate of OSA among adults is approximately 9–38%, of which approximately 13–33% occurs in males and 6–19% in females (Senaratna et al., 2017; Liguori et al., 2021). OSA can increase the risk of cardiovascular disease, hypertension, and sudden death and is often accompanied by psychiatric symptoms such as depression, anxiety, memory and cognitive impairment (Vanek et al., 2020). Neurocognitive impairment is an important clinical manifestation in patients with OSA, including decreased attention, alertness, memory, and executive dysfunction (Tahmasian et al., 2016; Bucks et al., 2017). Many previous studies have demonstrated that there is structural and functional damage and reduced cerebral perfusion in cognitive-related brain regions in patients with OSA, which are to some extent associated with sleep fragmentation and intermittent nocturnal hypoxemia (Torelli et al., 2011; Chen et al., 2017; Yan et al., 2021). However, at present, the exact neurological mechanism of cognitive impairment in OSA patients is still unclear.

Resting-state functional magnetic resonance imaging (rs-fMRI) is a common tool for the study of neuroimaging, and its widespread application is beneficial in improving our understanding of cognitive impairment in patients with OSA. Many studies have revealed the relationship between cognitive performance and alterations in different regions of the brain through structural and functional neuroimaging (Chen L.T. et al., 2018; Koo et al., 2020; Park et al., 2022). Regional homogeneity (ReHo) can be used to reflect the consistency of local neural activity between adjacent regions (Zang et al., 2004), and this analysis method has been proven effective in characterizing the local characteristics of brain activity and has been broadly used to localize functional abnormalities in brain disorders (Gupta et al., 2020; Lan et al., 2021). Our previous study showed abnormal ReHo values in the medial frontal gyrus, superior frontal gyrus, prefrontal lobe, angular gyrus, and left superior parietal lobule in patients with OSA. Some of these brain regions are considered to be linked to cognitive disorders such as episodic memory, executive cognitive control, and behavioral inhibition (Peng et al., 2014). Similar results have been found in studies by other teams (Santarnecchi et al., 2013; Zhou et al., 2020b; Song et al., 2022). These studies suggested that ReHo can be applied to localize abnormal brain regions in OSA patients. However, some literature has shown that brain activity at rest is a dynamic process and is related to specific functions (Zou et al., 2015; Ma X. et al., 2021). Resting-state ReHo assumes that the blood oxygen level-dependent signal is stable throughout the rs-fMRI scan while ignoring the characteristics of dynamic changes in brain activity over time (Yu et al., 2019). In recent years, some neuroimaging studies have shown that using the sliding window method to study the dynamic change in regional homogeneity, that is, dynamic regional homogeneity (dReHo), can provide a new perspective for understanding neural activity (Xue T. et al., 2020; Lu et al., 2021). This method has been applied to the study of depression (Yu et al., 2019; Xue K. et al., 2020), subacute stroke (Chen J. et al., 2018), trigeminal neuralgia (Yan et al., 2019), posttraumatic stress disorder (Fu et al., 2019), and several other diseases to explore the dynamic variations in local spontaneous neuronal brain activity. It has been shown that brain regions with large fluctuations in dReHo tend to be functional centers of the brain (Deng et al., 2016). All of the above studies demonstrate the application value of dReHo in neuroimaging. With this method, we can more accurately comprehend the dynamic process of local brain activity. Nevertheless, it remains unclear whether there are alterations in dReHo in patients with OSA.

In recent years, machine learning has been applied extensively to neuroimaging disease classification. Support vector machine (SVM) is one of the most widely used machine learning methods, which converts input data into high-dimensional space to find the optimal decision boundary to maximize the margins between datasets, allowing for highly precise individual-level classification and prediction (Perez-Riverol et al., 2017). Sun et al. employed SVM to classify individuals with bipolar disorder and unipolar disorder based on dReHo data and achieved an accuracy rate of 91.86% (Sun et al., 2021). Ma H. et al. (2021) also used this classifier to distinguish transient ischemic attacks from normal individuals with an accuracy rate of more than 80%. Thus, as SVM has been proposed as an effective tool for clinical diagnosis, we attempted to distinguish OSA patients from healthy controls (HCs) using SVM based on dReHo data.

Based on the above issues, we hypothesized that there is temporal variability in local spontaneous brain activity in patients with OSA, which is associated with cognitive dysfunction, and dReHo values could be regarded as a potential neural biomarker to differentiate OSA from HCs. To address the scientific hypothesis, we first studied changes in dReHo in OSA patients by using the sliding window approach (Chen J. et al., 2018) and explored its relationship with cognitive function. Second, by employing the SVM classification method, we further examined whether the altered dReHo could distinguish OSA patients from HCs.

## Materials and methods

### Participants

All participants in this study were recruited from the Department of Respiratory or Otorhinolaryngology Sleep Monitoring Room at the First Affiliated Hospital of Nanchang University from July 2013 to July 2021. According to the American Academy of Sleep Medicine criteria (Kapur et al., 2017), the inclusion criteria for OSA patients were as follows: (1) male; (2) apnea-hypopnea index (AHI) ≥ 15; (3) right-handed; and (4) between 18 and 60 years old. The inclusion criteria for HCs was an AHI of less than 5/h. The exclusion criteria for OSA patients and HCs were as follows: (1) hypertension, heart disease, or diabetes; (2) structural lesions (such as cysts or tumors) on brain MRI; (3) diseases of the central nervous system (such as neurodegenerative diseases, epilepsy, head injuries, psychosis, and current depression); (4) MRI contraindications (such as claustrophobia or metal implants or devices in the body); and (5) drug and psychotropic substance abuse. Finally, 81 untreated moderate or severe OSA patients and 84 HCs were recruited. The study was approved by the Medical Ethics Committee of the First Affiliated Hospital of Nanchang University, and all participants signed a written informed consent form in accordance with the Declaration of Helsinki.

### Polysomnography manuscript formatting

All participants underwent full nocturnal polysomnography (PSG) monitoring. The day before sleep monitoring, all participants were asked not to take hypnotic pills and drink alcohol or caffeinated beverages. Full nocturnal PSG was performed using the Respironics LE-Series Physiological Monitoring System (Alice 5 LE; Respironics, Orlando, FL, United States). At the same time, standard electroencephalogram (EEG), electroencephalogram (EOG), chin electromyography (EMG), electrocardiogram (ECG), thoracic and abdominal respiratory movements, and oxygen saturation (SaO_2_) were examined. Overnight PSG data of participants were recorded from 10:00 p.m. to 6 a.m. the next day. According to the guidelines of the American Academy of Sleep Medicine, obstructive apnea was defined as a decrease in airflow greater than or equal to 90% and lasting for more than 10 seconds; hypopnea was defined as a reduction in the airflow by 30% for at least 10 s, accompanied by a decrease in oxygen saturation greater than or equal to 3% and/or EEG awakening (Kapur et al., 2017). The AHI was calculated based on the average number of apnea and hypopnea events per hour during sleep. OSA was defined as an AHI ≥ 5/h, moderate OSA was defined as an AHI ≥ 15/h and < 30/h, and severe OSA was defined as an AHI > 30/h.

### Clinical and neuropsychological measures

All participants used the Epworth Sleepiness Scale (ESS, Chinese version) to assess excessive drowsiness during the day and the Montreal cognitive assessment (MoCA, Chinese version) scale to evaluate cognitive function. The ESS scale asked participants to rate their likelihood of falling asleep in an increasing probability from 0 to 3 in eight different situations. The highest score on the scale is 24, and a score greater than 6 indicates drowsiness, a score greater than 11 indicates excessive drowsiness, and a score greater than 16 indicates dangerous drowsiness. The MoCA scale consists of 8 cognitive items, including executive function, attention and concentration, abstract thinking, memory, language, visuospatial structure, calculation and orientation. The full score of the MoCA is 30, and a score < 26 indicates cognitive impairment. If the number of years of education is less than 12 years, one point will be added to adjust the educational deviation (Nasreddine et al., 2005). All of the above evaluations were conducted by a professionally trained doctor who did not know the clinical information of the participants. All the tests were carried out in the same order.

### Resting-state functional magnetic resonance imaging data acquisition

All participants used a 3.0T magnetic resonance system (Siemens, Erlangen, Germany) scanner with 8-channel phased-array magnetic head coils to collect MRI data in the First Affiliated Hospital of Nanchang University. In the scanning process, we used foam to reduce human-induced head movement and earplugs to reduce the noise interference of the scanner and asked all participants to relax, remain still, and close their eyes as much as possible without falling asleep and avoiding thinking as much as possible. First, all participants were arranged to collect routine T1-weighted and T2-weighted images to rule out brain structural lesions that might affect brain function or microstructure. Rs-fMRI data were obtained by a gradient echo plane imaging (EPI) sequence: repetition time (TR) = 2,000 ms, echo time (TE) = 30 ms, thickness = 4.0 mm, gap = 1.2 mm, field of view (FOV) = 230 × 230 mm^2^, flip angle = 90°, matrix = 64 × 64, slices = 30, duration 8 min, and 30 axial slices covering the whole brain. Finally, a magnetized fast gradient echo sequence (TR = 1,900 ms, TE = 2.26 ms, thickness = 1.0 mm, gap = 0.5 mm, FOV = 250 × 250 mm^2^, flip angle = 9°, resolution matrix = 256 × 256, and slices = 176) was used to obtain high-resolution three-dimensional T1-weighted brain structure MRI images. After the MRI scan, two senior radiologists examined for significant brain lesions, although no participants were excluded because of brain lesions.

### Data preprocessing

First, we checked the quality of all MRI images and eliminated incomplete brain images or artifacts using MRIcro software. Data Processing & Analysis for Brain Imaging (DPABI)^1^ software, based on MATLAB R2018b (MathWorks, Natick, MA, United States) platform and Statistical Parameter Mapping software package (SPM12)^2^ were used to preprocess fMRI data. During the processing, the first ten time points of each participant were removed to eliminate the effects of magnetic saturation and MRI scanner noise on the participants. Slice time correction and three-dimensional head motion correction were then performed on the remaining 230 volumes (Yan et al., 2013). If a participant had a maximum displacement of more than 1.5 mm in any direction during the entire fMRI scan or if the angle on any axis was rotated by more than 1.5°, that participant was excluded. The T1-weighted structure image was registered with the average value of the rearranged EPI image of each individual. Next, the new segmentation method in SPM 12 was used to segment the transformed structural image into gray matter, white matter, and cerebrospinal fluid. The functional data space of the participants was normalized to the Montreal Neurological Institute (MNI) template, and all images were subdivided into 3 × 3 × 3 mm^3^ voxels. Finally, time bandpass filtering (0.01–0.08 Hz) was used to reduce the effects of low-frequency drift, physiological high-frequency noise, and heart noise. Two subjects were excluded due to head movement of more than 1.5 mm.

### Dynamic regional homogeneity calculation

Dynamic regional index analysis was carried out using the DPABI-based temporal dynamic analysis (TDA) toolkit, and we applied this method to study the variability of dReHo. Ideally, the window size should be small enough to detect a potential instantaneous signal but large enough to analyze the lowest frequency of interest in the signal (Sakoğlu et al., 2010; Chen J. et al., 2018). To avoid introducing false fluctuations, the minimum window length should be larger than 1/f_*min*_, where f_*min*_ is the minimum frequency of the time series (Leonardi and Van De Ville, 2015; Fu et al., 2019). Because there is no consensus on the choice of window length and step size, according to previous research, we chose the medium-length sliding window size of 30 TR to capture brain dynamics and set the window step size of sliding over time to 1 TR (Yan et al., 2019). Then, the ReHo graph of each sliding window is calculated to estimate the dReHo of all windows. Next, we divided the mean ReHo of the whole brain to normalize the ReHo map z transformation of each voxel. Finally, the Gaussian check dReHo map with a full-width at half-maximum (FWHM) of 6 mm was used for smoothing.

### Statistical analyses

The Kolmogorov–Smirnov test was used to test the normality of the clinical data. The demographic and clinical data of the two groups were analyzed by independent sample *t*-test tests with IBM Statistical Package for the Social Sciences (SPSS) V 26.0 statistical software (Armonk, NY, United States). Taking head movement parameters, years of education, and age as covariates, a double-sample *t*-test using Data Processing & Analysis for Brain Imaging (DPABI; see text footnote 1) statistics based on MATLAB R2018b (MathWorks, Natick, MA, United States) was used to test the difference in the dReHo map between OSA patients and HCs on each voxel. The multilevel comparison was corrected by Gaussian random field theory (GRF, double tail, voxel-level *P* < 0.01 and cluster level *P* < 0.05). Finally, Pearson correlation analysis was carried out between the mean dReHo z value of abnormal brain areas and clinical data in patients with OSA. *P* < 0.05 was considered to be statistically significant.

### Support vector machine analysis

To test whether the dReHo value could be used to distinguish OSA patients from HCs, the difference value of dReHo between groups was used as the classification feature, and then the Sklearn.svm.LinearSVC() in Python was used for SVM analysis, with all parameters set to default values. After that, we trained the SVM by providing labeled observations with known classification results. To overcome the limitations of our samples, we applied the leave-one-out cross-validation method to estimate the generalization ability of our classifier (Chen J. et al., 2018). Finally, the accuracy, sensitivity, and specificity were obtained to evaluate the performance of the classifier.

### Validation analysis

To further test the reliability of our dReHo results, we reanalyzed the rs-fMRI data using two additional window lengths (25 TR and 35 TR).

## Results

### Demographic and clinical characteristics of participants

The demographic and clinical data for both groups conformed to a normal distribution, and the detailed values are shown in Table 1. We regressed the age and the years of education as covariates. Compared to the HCs, the body mass index (BMI), AHI, sleep period time (SPT), oxygen desaturation index (ODI), SaO_2_ < 90% and ESS score of OSA patients were significantly higher, while sleep efficiency, mean SaO_2_, nadir SaO_2_, the total score of MoCA and other subdivisions were significantly lower. There was no significant difference in total sleep time and naming score of the MoCA scale between OSA patients and HCs (*P* > 0.05).

**TABLE 1:** Demographic and clinical data between OSA patients and HCs.

Characteristic	OSA patients (*N* = 79)	HCs (*N* = 84)	*t*-value	*P*-value
Age, years	38.5 ± 9.3	45.3 ± 12.1	−4.06	<0.001
Education, years	12.8 ± 3.0	11.2 ± 3.1	3.43	0.001
BMI, kg/m2	27.2 ± 3.5	21.1 ± 1.7	14.04	<0.001
Total sleep time, min	387.5 ± 98.4	407.3 ± 22.9	−1.75	0.084
Sleep period time, min	460.9 ± 71.6	441.8 ± 22.3	2.27	0.026
Sleep efficiency, %	83.6 ± 17.8	92.2 ± 3.5	−4.23	<0.001
AHI/hour	54.0 ± 21.7	2.2 ± 1.2	21.19	<0.001
SaO_2_ < 90%	41.2 ± 48.1	0.63 ± 1.3	7.50	<0.001
Nadir SaO_2_, %	68.1 ± 12.4	93.8 ± 3.6	−1.72	<0.001
Mean SaO_2_, %	92.0 ± 4.1	96.9 ± 2.1	−9.34	<0.001
ODI	49.9 ± 24.6	1.8 ± 1.1	17.39	<0.001
MoCA, scores	24.4 ± 3.3	28.0 ± 1.4	−9.06	<0.001
MoCA:visual space and execution	3.9 ± 0.9	4.7 ± 0.5	−6.41	<0.001
MoCA:naming	2.9 ± 0.3	2.9 ± 0.2	−1.30	0.197
MoCA:delayed memory	2.6 ± 1.5	4.3 ± 0.6	−9.59	<0.001
MoCA:attentional function	5.1 ± 1.3	5.5 ± 0.5	−2.88	0.005
MoCA:language	2.2 ± 0.8	2.7 ± 0.4	−5.50	<0.001
MoCA:abstract	1.5 ± 0.5	1.9 ± 0.2	−6.76	<0.001
MoCA:orienteering	5.7 ± 0.7	5.9 ± 0.3	−2.13	0.035
ESS, scores	11.5 ± 4.4	1.4 ± 1.3	19.48	<0.001

OSA, obstructive sleep apnea; HCs, healthy controls; N, number; BMI, body mass index; AHI, apnea-hypopnea index; SaO_2_, oxygen saturation; SaO_2_ < 90%, percentage of total sleep time spent at oxygen saturation less than 90%; ODI, oxygen desaturation index; MoCA, Montreal Cognitive Assessment; ESS, Epworth Sleepiness Scale.

### Differences in dynamic regional homogeneity between the obstructive sleep apnea patients and healthy controls

**FIGURE 1:**
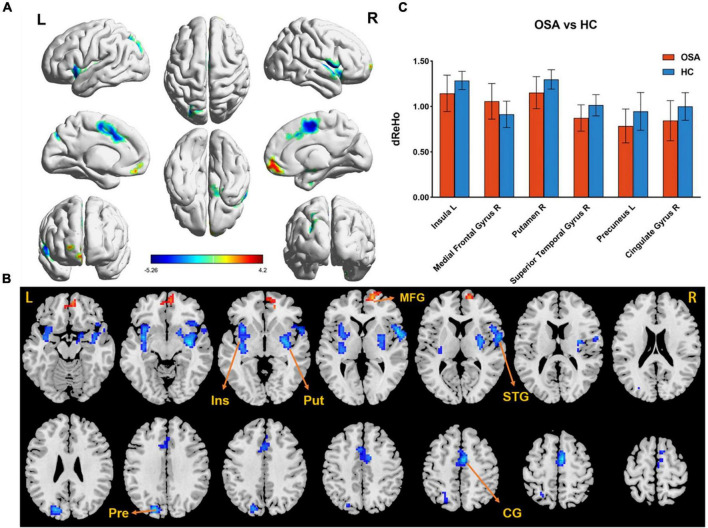
**(A,B)** The differences in dReHo between the OSA patients and HCs (after GRF correction voxel-wise *p* < 0.01, cluster-wise *p* < 0.05, two-tailed). The color bar indicates the *t*-value. **(C)** The histogram indicates mean value of dReHo variability between the two groups. Detailed information about these altered regions are described in Table 2. L, left; R, right; dReHo, dynamic regional homogeneity; Ins, Insula; Put, Putamen; MTG, Medial Frontal Gyrus; STG, Superior Temporal Gyrus; Pre, Precuneus; CG, Cingulate Gyrus.

The difference in dReHo between the two groups is shown in Table 2 and Figure 1. Compared with HCs, OSA patients had significantly lower dReHo in the right putamen, right superior temporal gyrus, right cingulate gyrus, left insula, and left insula, while dReHo in the right middle frontal gyrus was significantly higher in OSA patients than in HCs. All results were reported at voxel-level *P* < 0.01 and cluster-level *P* < 0.05 and were GRF corrected.

**TABLE 2:** Brain regions with significant differences in dReHo between OSA patients and HCs.

Brain regions	BA	Voxel	MNI coordinates of peak voxel			*t-value*
			X	Y	Z	
Right medial frontal gyrus	/	150	15	60	6	4.1992
Left insula	13	356	−33	12	−3	−4.6593
Right putamen	/	376	33	−9	−6	−5.1785
Right superior temporal gyrus	22	260	54	9	3	−4.8262
Left precuneus	/	158	−18	−81	33	−4.8002
Right cingulate gyrus	/	307	3	−6	54	−5.2614

All clusters were reported with a voxel-level threshold of *P* < 0.01, and cluster-level of *P* < 0.05, two tailed, GRF correction. BA, Brodmann area; MNI, Montreal Neurological Institute; dReHo, dynamic regional homogeneity.

### Correlational analysis results

**FIGURE 2:**
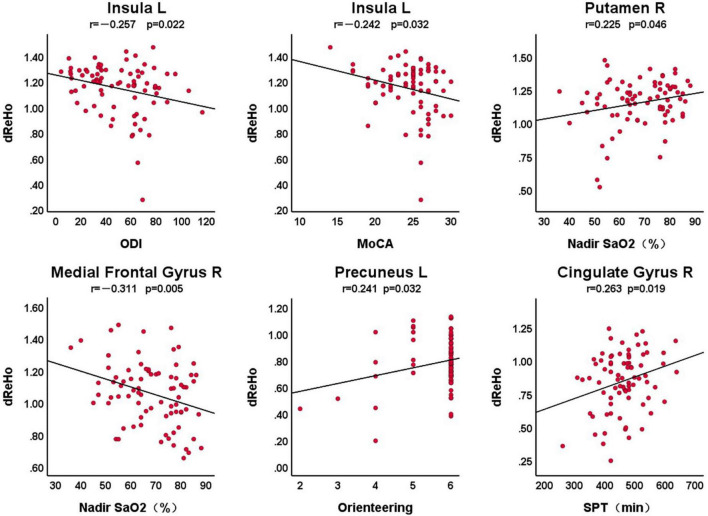
The correlation between dReHo in abnormal brain areas and clinical variables in patients with OSA. L, left; R, right; dReHo, dynamic regional homogeneity; MoCA, Montreal Cognitive Assessment; ODI, oxygen desaturation index; SPT, sleep period time.

Figure 2 shows the correlation between dReHo in abnormal brain areas and clinical variables in patients with OSA, with a statistically significant difference of *P* < 0.05. We found that there was a significant correlation between the left insula and ODI (*r* = –0.257, *P* = 0.022), the total score of MoCA (*r* = –0.242, *P* = 0.032), the right putamen and nadir SaO_2_ (*r* = 0.225, *P* = 0.046), the right medial frontal gyrus and nadir SaO_2_ (*r* = –0.311, *P* = 0.005), the left precuneus and the orientation of the MoCA scale (*r* = 0.241, *P* = 0.032), and the right cingulate gyrus with the SPT (*r* = 0.263, *P* = 0.019).

### Support vector machine analysis results

**FIGURE 3:**
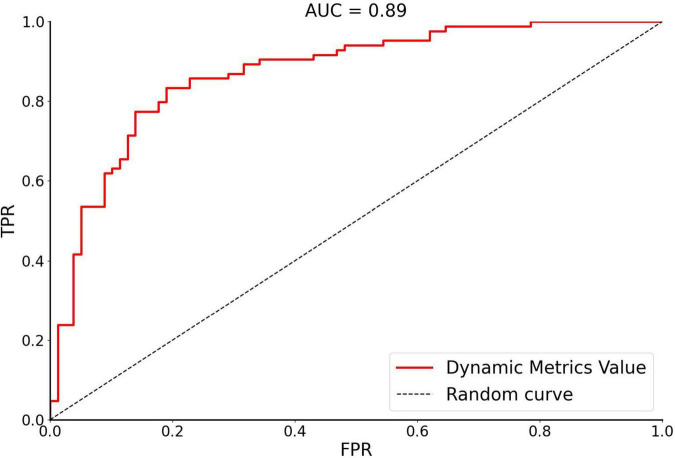
The receiver operating characteristic curve of dynamic metrics. The image of ROC was displayed using Python software. Accuracy of 81.60%, Precision of 81.01%, Sensitivity of 81.01%, Specificity of 82.14%, and area under the curve (AUC) of 0.89. FPR, false positivity rate; TPR, true positivity rate; AUC, area under the receiver operating characteristic curve.

In this study, SVM was used to calculate the accuracy, precision, sensitivity, specificity, and receiver operating characteristic (ROC) curve of the classifier (Figure 3). The curve was drawn by Python software. For OSA patients and HCs, the performance of the classifier achieved an accuracy of 81.60%, precision of 81.01%, sensitivity of 81.01%, specificity of 82.14%, and area under the curve of 0.89.

### Validation results

The validation analysis shows that when different window lengths are used, the results produced are roughly the same as our main finding using a 30 TR window width. The results for different window lengths (25 TR and 35 TR) are shown in Supplementary Figures 1, 2.

## Discussion

The present study is the first to employ dReHo to examine the changes in internal brain activity in OSA patients. Compared with HCs, OSA patients had higher dReHo values in the right medial frontal gyrus and had a significant negative correlation with the nadir SaO_2_. In contrast, the dReHo values were lower in the right putamen, right superior temporal gyrus, right cingulate gyrus, left insula and left precuneus, and these brain regions were significantly correlated with the MoCA score, ODI, nadir SaO_2_ and SPT. In addition, we considered the dReHo values of abnormal brain dynamic areas as classification characteristics in this study and obtained an SVM classification accuracy of 81.60%, precision of 81.01%, sensitivity of 81.01%, specificity of 82.14%, and AUC of 0.89. In general, these findings provide evidence for dynamic local abnormalities in patients with OSA, which may contribute to further understanding of the neurophysiological basis of the disease and establish objective neurobiomarkers.

According to the study of Zhang et al. (2013) it is known that the change in the rs-fMRI signal is related to the influence of OSA disease. Studies have shown that obesity is one of the important risk factors for OSA patients (Drager et al., 2013). The BMI of OSA patients in this study was higher than that of HCs. Previous studies have found that abnormal functional connectivity between the hippocampus and caudate nucleus may be caused by OSA itself rather than obesity (Song et al., 2018). Although there was a difference in BMI between OSA patients and HCs in this study, we performed regression analysis as a covariate to control for the effect of this factor. Therefore, we concluded that the difference in dynamic local consistency in the present study was caused by OSA.

The dReHo values in the left precuneus and the right cingulate gyrus of OSA patients in this study were reduced compared to HCs, and the dReHo values in the right medial frontal gyrus were increased compared to those of HCs. The above brain regions are essential components of the default mode network (DMN) (Li et al., 2019), which was first described by Raichle et al. (2001) and involves a range of advanced cognitive functions, such as visuospatial imagery, consciousness, attention, episodic memory, executive cognitive control, and behavioral inhibition (Andrews-Hanna et al., 2010; Andrews-Hanna, 2012). Both the precuneus and the posterior cingulate gyrus belong to the posterior DMN, which are primarily responsible for cognitive processing and memory extraction (Coutinho et al., 2016; Li et al., 2019). Previous studies have also found structural and functional abnormalities in the above brain regions in OSA patients (Joo et al., 2013; Li et al., 2016; Lee et al., 2019). In the current study, the dReHo of the precuneus and cingulate gyrus decreased in patients with OSA, and the left precuneus was positively correlated with the orienting component of the MoCA scale. Previous studies have also revealed that the core region of the precuneus is activated for multiple orienting domains (Peer et al., 2015), so we hypothesized that this might be a potential mechanism for cognitive impairment in OSA patients. In a study by Baril et al. (2017), hypoxemia was discovered to be associated with a core component of the default network, further speculating that cognitive impairment in OSA patients may be connected to hypoxic damage to precuneus function. Our former studies and others have shown decreased left precuneus ReHo values in patients with OSA, which is analogous to our results (Santarnecchi et al., 2013; Ji et al., 2021). In our study, the right cingulate gyrus had a weak positive correlation with SPT, and we speculate that the decrease in SPT may be the result of cingulate gyrus damage. The medial frontal gyrus, an important element of the prefrontal lobe, also participates in the composition of the DMN, which plays a critical role in memory, cognition, decision-making, social behavior, and emotion (Klune et al., 2021). A previous study discovered that ReHo in the medial frontal gyrus was decreased in OSA patients and was negatively correlated with cognitive impairment (Zhou et al., 2020b). Structural damage in this brain region has been found in other studies in OSA patients (Chan et al., 2014; Tummala et al., 2016). The dReHo values in the right medial frontal gyrus were increased in OSA patients in this study compared to HCs and negatively correlated with the nadir SaO_2_. Earlier studies have demonstrated that the frontal cortex is vulnerable to sleep disruption and intermittent hypoxia (Beebe and Gozal, 2002); hence, we speculate that this may be due to a hypoxia-induced functional compensatory response in this brain region.

The insula is a vital hub in the salience network that is involved in somatosensory, autonomic, emotional, and cognitive processing (Borsook et al., 2016), and plays an essential role in switching between the central executive network and the DMN (Tahmasian et al., 2016). The right insula is in charge of the sympathetic nerve, and the left insula is mainly responsible for the parasympathetic nerve, which lowers blood pressure and heart rate, and this area interacts with the hypothalamus and contributes to the control of the sleep cycle (Oppenheimer et al., 1992; Tummala et al., 2016). It has been found that thinning of the left insular cortex may facilitate increased sympathetic activity in OSA patients by reducing the effect on the normal insula of the hypothalamus (Macey et al., 2018). The increased sympathetic activity induced by chronic intermittent hypoxia in OSA patients may lead to neurocognitive deficits by eliciting inflammation in organs and vascular beds (Idiaquez et al., 2014). Our study showed decreased dReHo in the left insula, and previous studies have also suggested that local cortical thinning of the insula (Joo et al., 2013), as well as diminished mean diffusivity values (Kumar et al., 2012), were found in patients with OSA. Consequently, we hypothesize that nocturnal intermittent hypoxemia may lead to structural and functional impairment of the insula, which in turn leads to neurocognitive deficits. We noticed that the left insula was negatively correlated with ODI and the total scores of MoCA. This indicates that with the increase in the severity of the disease, the damage to this brain region becomes more serious, further confirming our speculation.

Compared with that of HCs, the dReHo in the right putamen of OSA patients in this study was also significantly lower. The putamen regulates autonomic nerve movement and cognitive, memory, emotional, language, and motivational learning (Kumar et al., 2014; Uono et al., 2017). A former functional MRI study revealed increased neural activity and functional connectivity in the putamen during autonomic nerve stimulation (Song X. et al., 2019). To our knowledge, a major signature of OSA is impaired regulation of the autonomic nervous system (that is, derangement of upper airway regulation) (Harper et al., 2003). In the present study, we discovered reduced dReHo in the right putamen of OSA patients. We speculate that this is possibly caused by intermittent hypoxemia at night in patients with OSA, resulting in putamen damage and leading to autonomic motor disorders and neurocognitive dysfunction. It has also been previously reported that upper airway dysregulation in patients with OSA may be due to structural injury in the putamen region (Kumar et al., 2014). In addition, the dReHo value of the putamen was positively correlated with the nadir SaO_2_ to a certain extent, indicating that the dReHo of the putamen decreased, and the nadir SaO_2_ amount also declined. This further confirms our speculation about the possible mechanism of impaired autonomic movements in OSA patients.

Damage to the superior temporal gyrus, an auditory language center, can cause receptive aphasia and may play a role in emotional processing as well as social cognition (Song L. et al., 2019; Liu et al., 2021). It has been proposed that the nerve response of the superior temporal gyrus in patients with OSA is weakened, which probably contributes to the prolongation of apnea in OSA (Henderson et al., 2003) because the superior temporal gyrus is activated in the sensation and movement of the upper airway (Martin et al., 2001). Earlier studies have observed reduced local brain activity and functional connections in the right superior temporal gyrus of OSA patients (Santarnecchi et al., 2013; Zhou et al., 2020a), and previous studies by our group have also illustrated that ReHo in the right superior temporal gyrus decreased and that the ReHo value increased after continuous positive airway pressure treatment (Li et al., 2021). The abnormal functional connectivity of the superior temporal gyrus in OSA patients was confirmed to be associated with neurocognitive deficits in a study by Zhou et al. (2020a). With the decreased dReHo in the right superior temporal gyrus of OSA patients in our study, we hypothesize that the intermittent hypoxia arising from abnormal upper airway movements caused by injury to the superior temporal gyrus is likely to influence cognitive function. This might provide a new perspective for understanding the mechanisms of cognitive impairment in OSA patients.

SVM is a type of machine that learns algorithms to search for diagnostic biomarkers of OSA and may improve the accuracy of diagnosis and the precision of treatment. Moreover, with the intensive development of machine learning technology, it will be a general trend for doctors to utilize artificial intelligence to diagnose and manage the health of patients. Moreover, SVM has been extensively applied to various diseases and has achieved good classification performance (Gui et al., 2021; Ma H. et al., 2021). Using an SVM classifier, OSA patients can be distinguished from HCs by dynamic local indicators. This study achieves a high recognition accuracy of 85.88% between OSA patients and HCs. The results may indicate the potential value of dynamic local indices in the clinical diagnosis of OSA.

Comparing previous studies, we noticed that the results of dynamic measurements overlap with those of static measurements, which are similar to other disease studies (Córdova-Palomera et al., 2017; Yu et al., 2019). Previous studies have revealed that the ReHo values of the insula and putamen in OSA patients were relatively elevated (Peng et al., 2014; Song et al., 2022), while the ReHo of the medial frontal gyrus was comparatively decreased (Zhou et al., 2020b), which differs from our findings, and we suppose that this may be attributed to population differences in subjects and variations in dynamic metrics.

## Limitations

Several limitations exist in this study. First, this study recruited moderate and severe male OSA subjects, which to some extent influenced our ability to generalize the findings to the total population with this disease. Future studies should include patients with mild OSA and female patients for further study. Second, although the sample size is relatively large compared with other studies, it is not large enough, which may limit the representativeness of the results. Third, we only explored the changes in dynamic brain activity, but the alterations of whole-brain dynamic functional connections are not clear, and future studies are required to investigate the changes in whole-brain dynamic functional connectivity in OSA patients. Finally, because obesity is an important risk factor for OSA, the BMI values of the OSA patients in this study were higher than those of the HCs, and although BMI was used as a covariate in the analysis, the BMI values may have biased the current findings and were further refined in the follow-up study.

## Conclusion

In the present study, we used a sliding window approach to calculate dReHo to investigate the characteristics of dynamic changes in local brain activity in OSA patients. We observed that spontaneous brain activity abnormalities with dynamic characteristics existed in OSA patients and were related to neurocognition, while SVM based on dReHo values of abnormal brain regions could distinguish OSA from normal individuals. This is likely to provide essential evidence for comprehending the potential neuropathology of OSA while providing a possible neuroimaging marker for clinical diagnosis.

## Data availability statement

The raw data supporting the conclusions of this article will be made available by the authors, without undue reservation.

## Ethics statement

The studies involving human participants were reviewed and approved by the Ethics Committee of the First Affiliated Hospital of Nanchang University. The patients/participants provided their written informed consent to participate in this study. Written informed consent was obtained from the individual(s) for the publication of any potentially identifiable images or data included in this article.

## Author contributions

DP guided and designed the MRI experiment. YS and PY analyzed the resting-state fMRI data. KL and YS analyzed and discussed the ideas of the manuscript. KL organized the results and wrote the manuscript. KL, XL, WX, PL, LK, YZ, LH, LZ, and TL collected the resting fMRI data and applied for ethics approval. HL and DP reviewed and revised the manuscript. All authors contributed to the article and approved the submitted version.

## Footnotes

1
http://rfmri.org/dpabi

2
https://www.fil.ion.ucl.ac.uk/spm/software/spm12/
