# Supplementary material for: Dynamic regional homogeneity alterations and cognitive impairment in patients with moderate and severe obstructive sleep apnea
Source: Front Neurosci. 2022 Aug 26;16:940721. doi: 10.3389/fnins.2022.940721 (PMC9459312; doi:10.3389/fnins.2022.940721)
Supplement: Supplementary file 1 [file Data_Sheet_1.docx]

Supplementary Material

## Supplementary Figures


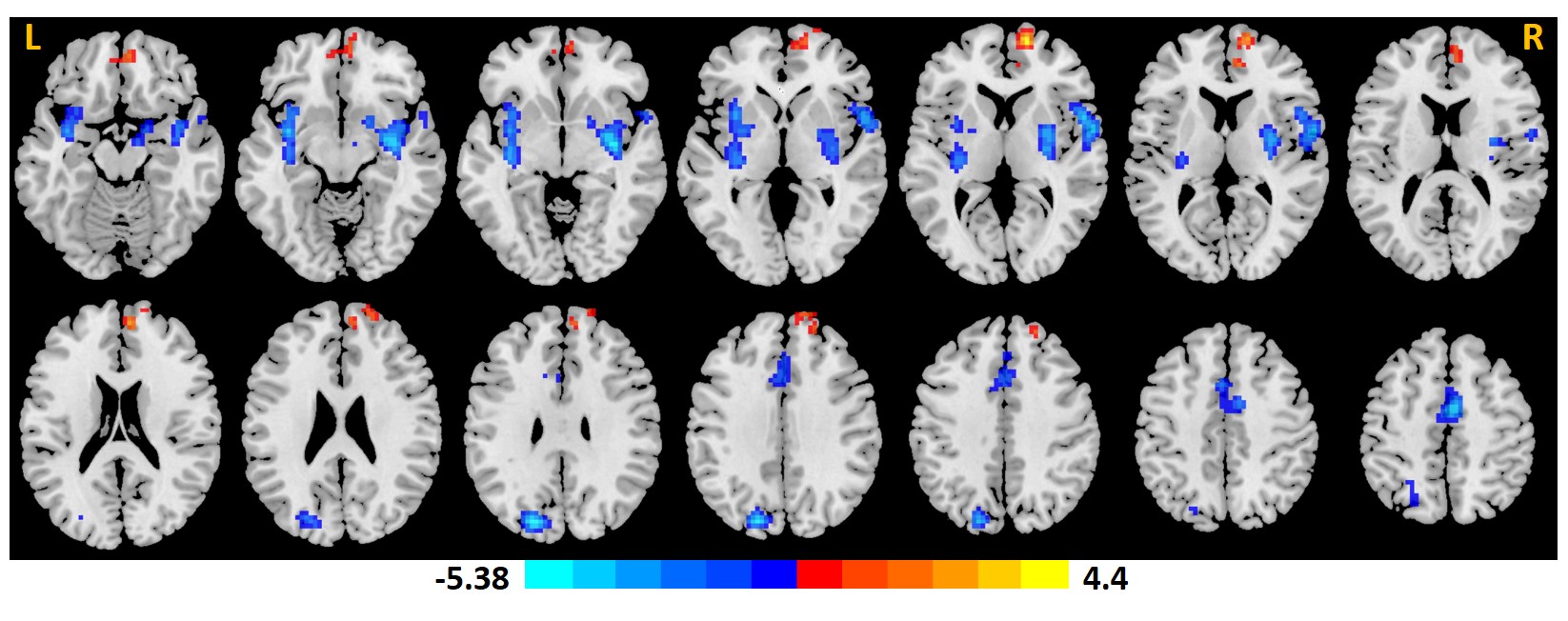


**Supplementary Figure S1.** Brain regions with significantly different dReHo between OSA and HCs at a window length of 25 TR. (GRF-corrected; voxel-wise p<0.01, cluster-wise p<0.05, two-tailed). Color bars indicate T values. L, left; R, right; dReHo, dynamic regional homogeneity.


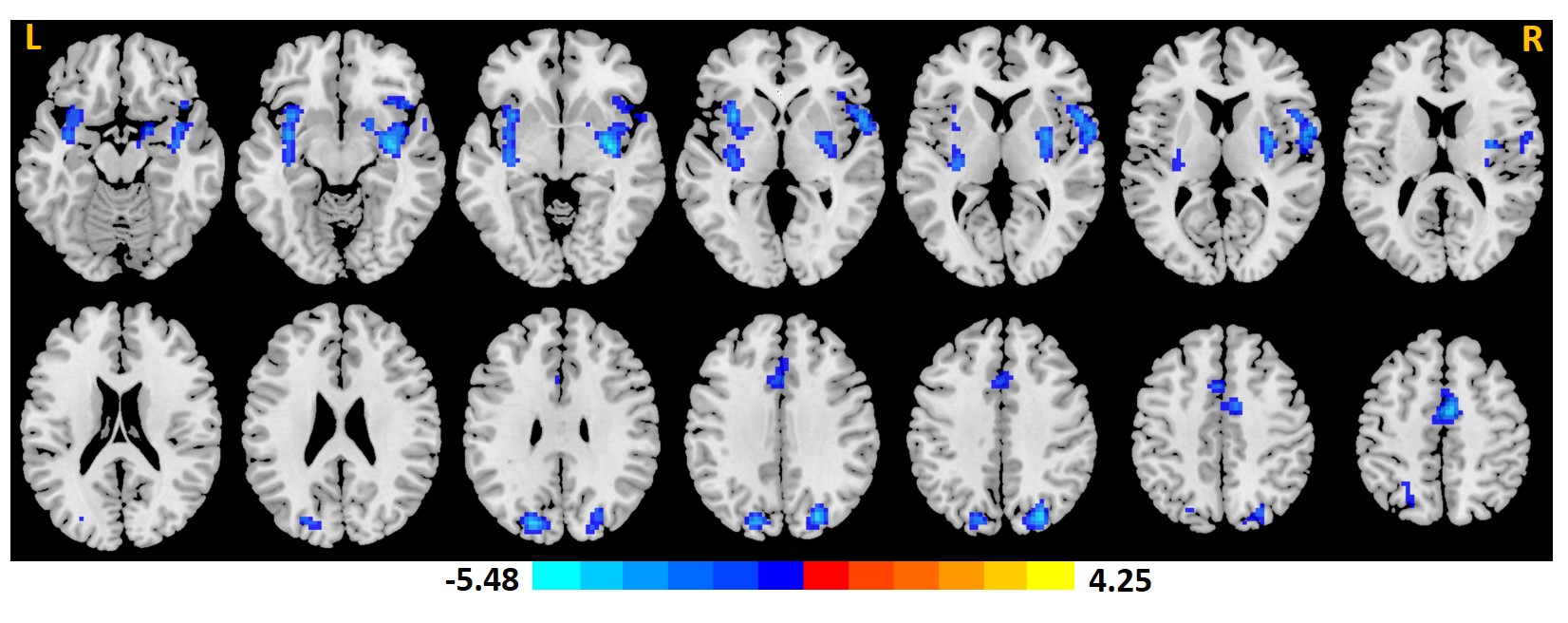


**Supplementary Figure S2.** Brain regions with significantly different dReHo between OSA and HCs at a window length of 35 TR. (GRF-corrected; voxel-wise p<0.01, cluster-wise p<0.05, two-tailed). Color bars indicate T values. L, left; R, right; dReHo, dynamic regional homogeneity.
